# The Place of Obsessive-Compulsive and Related Disorders in the Compulsive-Impulsive Spectrum: A Cluster-Analytic Study

**DOI:** 10.1017/S109285292100033X

**Published:** 2021-04-12

**Authors:** Leonardo F Fontenelle, Louise Destrée, Mary-Ellen Brierley, Emma M Thompson, Murat Yücel, Rico Lee, Lucy Albertella, Sam R Chamberlain

**Affiliations:** 1 Turner Institute for Brain and Mental Health, Monash University, 770 Blackburn Road, Clayton, Victoria 3168, Australia; 2 Obsessive, Compulsive, and Anxiety Spectrum Research Program. Institute of Psychiatry, Federal University of Rio de Janeiro (UFRJ) & D’Or Institute for Research and Education (IDOR), Rio de Janeiro, Brazil

**Keywords:** Compulsivity, Impulsivity, Obsessive-Compulsive Disorder, Body Dysmorphic Disorder, Hoarding Disorder, Trichotillomania, Neurodermatitis

## Abstract

**Objective:**

The extent to which obsessive-compulsive and related disorders (OCRDs) are impulsive, compulsive or both requires further investigation. We investigated the existence of compulsive, impulsive, and compulsive-impulsive clusters in an online non-clinical sample and in which groups DSM-5 OCRDs and other related psychopathological symptoms are best placed.

**Methods:**

774 adult participants completed online questionnaires including the Cambridge-Chicago Compulsivity Trait Scale (CHI-T), the Barratt Impulsiveness Scale (BIS-11), and a series of DSM-5 OCRDs symptom severity and other psychopathological measures. We used K-means cluster analysis using CHIT and BIS responses to test three and four factor solutions. Next, we investigated whether different OCRDs and other psychopathological symptoms predicted cluster membership using a multinomial regression model.

**Results:**

The best solution identified one “healthy” and three “clinical” clusters (i.e. one predominantly “compulsive” group, one predominantly “impulsive” group, and one “mixed – “compulsive and impulsive group”). A multinomial regression model found obsessive-compulsive, body dysmorphic, and schizotypal symptoms to be associated with the “mixed” and the “compulsive” clusters, and hoarding and emotional symptoms to be related, on a trend level, to the “impulsive” cluster. Additional analysis showed cognitive-perceptual schizotypal symptoms to be associated with the “mixed” but not the “compulsive” group.

**Conclusions:**

Our findings suggest that obsessive-compulsive disorder; body dysmorphic disorder and schizotypal symptoms can be mapped across the “compulsive” and “mixed” clusters of the compulsive-impulsive spectrum. Although there was a trend towards hoarding disorder symptoms being associated with the “impulsive” group, trichotillomania and skin picking disorder symptoms did not clearly fit to the demarcated clusters.

## Introduction

According to the Diagnostic and Statistical Manual of Mental Disorders (DSM-5)^1^ and the 11^th^ revision of the International Classification of Diseases, ^2^ obsessive-compulsive and related disorders (OCRDs) comprise a group of disorders that share repetitive thoughts and/or behaviors, key diagnostic validators, and underlying etiology. They include obsessive-compulsive disorder (OCD), body dysmorphic disorder (BDD), hoarding disorder (HD), trichotillomania (TTM; hair pulling disorder), and excoriation (skin picking) disorder (SPD) in the DSM-5. ^1^ Yet, despite not having official status as an OCRD in the current classification schemes, many other conditions seem to share characteristics with OCD. We are not aware of epidemiological studies considering the broad prevalence and impact of OCRDs as a group, but based on existing studies and comorbidity rates, we estimate that more than 15% of the general populations exhibit at least one current OCRD. ^3–7^ Therefore, their societal and economic impacts are probably colossal. ^8^

The idea that these disorders could be somehow related to each other was initially systematized by Hollander and Wong, ^9^ who described a unidimensional spectrum of conditions characterized by an inability to inhibit or delay behaviors. This spectrum would display on one end, compulsive (risk aversive) disorders (including OCD, BDD, anorexia nervosa, and hypochondriasis, among others) and on the other end, impulsive (risk seeking) conditions (such as self-injurious behaviors, pathological gambling, kleptomania, and compulsive buying). Based on studies showing failures within cortico-striatal neurocircuitry that regulate inhibitory control as a common features across these disorders Fineberg et al. ^10^ advanced Hollander and Wong’s ^9^ model by suggesting that, instead of lying in a unidimensional model, compulsivity and impulsivity are overlapping but are also partly dissociable constructs that share a complex relationship. ^9,11–13^ Thus, in this new model, these disorders could fall into a predominantly compulsive, a predominantly impulsive or a mixed compulsive-impulsive cluster.

It is often believed that compulsivity and impulsivity traits reflect a propensity to develop OCRDs or addictions at some point in life; thus, being candidate vulnerability markers. ^14^ Yet, precise information on the underpinnings of compulsivity and impulsivity in OCRDs is lacking. One connectivity study found the neural correlates of compulsivity and impulsivity to be independent of DSM-IV OCD and gambling disorder diagnoses, ^15^ thus suggesting that circuits underlying these functions could be pursued as a biological basis of the compulsive-impulsive cluster. Further, experts in OCRDs and addictions have independently argued that systems governing habit, inhibitory control, and compulsivity are important constructs for both OCRDs and addictions. ^16,17^ On the other hand, the constructs that differentiated OCRDs from addictions included performance monitoring in OCRDs ^16^ and reward valuation, reward expectancy, action selection, reward learning in addictions. ^17^ Yet, few studies have focused on the clinical facets of compulsivity and impulsivity and how they relate to each other.

An important, but often-neglected aspect of this discussion is what researchers mean by impulsivity and compulsivity. Behaviorally, impulsivity has been defined as “actions that are poorly conceived, prematurely expressed, unduly risky, or inappropriate to the situation and that often result in undesirable outcomes”. ^18^ However, there is still debate on the best definition of compulsivity. For instance, Luigjes et al. ^19^ listed *phenomenological aspects* (e.g. the subjective experience that one “has to” perform a behavior), *observational characteristics* (e.g. the repetitiveness, non adaptiveness, inappropriateness, or the habitualness of a behavior) and *explanatory* functions (such as its’ distress-reducing objective) as compulsivity features in different literature descriptions. ^19^ Thus, a compulsive-impulsive cluster of different traits can be relatively heterogeneous to accommodate more than one OCRD phenotype. A recent review noted that the Barratt Impulsiveness Scale-11 (BIS-11) and the Cambridge-Chicago Compulsivity Trait Scale (CHI-T) are suitable to measure impulsivity and compulsivity as transdiagnostic traits that are relatively free of the influence of discrete and co-existing symptoms/syndromes. ^14^

Research on the underlying traits associated with OCRDs can provide insights for prevention and identification of individuals at risk for OCRDs. Yet, there are still several questions pertaining to the symptomatic organization of the compulsive-impulsive spectrum, including the place of different disorders within the model. That said, we investigated (1) the existence of a “compulsive”, an “impulsive”, and a “compulsive-impulsive” cluster in an online non-clinical sample and (2) where DSM-5 OCRDs symptoms could be placed within these clusters. We hypothesized that increased OCD symptoms would be more likely to originate from a “compulsive” cluster (for representing a prototypical compulsive disorder^20^); that greater severity of TTM and SPD would be more likely to develop from the impulsive group (for being traditionally classified as impulse control disorders^20^; and that increased HD and BDD symptoms severity would more likely to originate from a “compulsive- impulsive” cluster (as many of the typical symptoms of these disorders may be construed as poor impulse control). Importantly, we also predicted that the association between BDD and HD symptoms and the compulsive-impulsive cluster would be independent of the lower insight or psychotic-like/schizotypal features that are frequently reported in individuals with these conditions.^21^

## Methods

### Participants

Adult participants (≥ 18 years of age) were recruited for this cross-sectional study through Amazon Mechanical Turk (an American online crowdsourcing platform). The advertisement for the study was made available to all workers on the platform who resided in the United States, were over the age of 18, and had English as their first language or learnt English before the age of 7 (as all questionnaires were in English). The study was advertised as an investigation on the relationship between day-to-day behaviors, lifestyle, life experiences and wellbeing. Interested participants were directed to a Qualtrics-based series of questionnaires, where informed consent was given.

The survey took approximately 90 minutes to complete, after which time participants received a code to be entered in the Mechanical Turk website to be reimbursed US$15. Participants could leave the survey and come back within 24 hours to complete it. Yet, to maximize the validity of the survey results, individuals could not attempt the survey twice. All study procedures were carried out in accordance with the Declaration of Helsinki, and participants provided informed consent. The Monash University Human Research Ethics Committee ethically reviewed and approved the study.

### Assessment

#### Compulsivity

The CHI-T is a self-report scale that covers the compulsivity construct with 15 broad statements to which subjects agree/disagree in different levels, such as preference for more immediately rewarding activities, and the need for completion^22^ Response to each item ranges from strongly disagree (0) to strongly agree (3). Total scores range from 0 to 45 with higher scores indicating higher compulsivity. An initial factor analysis study indicated the presence of one factor related to reward-seeking/need for perfection, and another related to the anxiolytic/soothing features of compulsivity. For the purposes of the present study, only the total CHI-T score was used. The CHI-T has shown excellent psychometric properties.^22^

#### Impulsivity

The Barratt Impulsivity Scale (BIS-11) is a 30-item self-report scale that measures trait impulsivity, i.e. the individual’s tendency to think and behave impulsively across a range of situations^23^. The participant must assess whether each item (e.g. “I plan tasks carefully”) applies to him/her and rate them according to a Likert scale ranging from 1 (rarely or never) to 4 (almost always/always). The scale total score ranges from 0 to 120, with a higher score indicating greater impulsivity. Although the BIS-11 has subscales addressing motor, attentional, and non-planning impulsivities, we were specifically interested in the BIS-11 total scores to maintain simplicity of the statistical models and clarity of interpretation. The BIS-11 has shown appropriate psychometric properties^24^.

#### Obsessive-Compulsive Symptoms

The Dimensional Obsessive-Compulsive Scale (DOCS) is a 20-item self-report questionnaire that evaluates the severity of the four most reliably replicated dimensions of OCD symptoms including contamination, fear of harm, unacceptable thoughts, and symmetry. For each symptom dimension, five different features (time spent, avoidance, distress, interference and control) are assessed and measured on a scale ranging from 0 to 4 ^25^. Subscale scores are obtained by summing the five items of each subscale (range = 0-20), which are summed to obtain total score (range = 0-80)^25^, with a higher score indicating more severe OCD symptoms. Initial regression models considered the total DOCS scores. The DOCS has demonstrated excellent psychometric characteristics. The DOCS’s cut-off score is 21.

#### Body Dysmorphic Symptoms

The Appearance Anxiety Inventory (AAI) is a 10-item self-report tool to quantify the severity of the responses to a distorted body image, particularly avoidance behavior and threat monitoring (e.g. “I compare aspects of my appearance to others”). ^26^ Participants are asked to select the response that best describes the way they felt about the appearance of a specific feature over the past week, with responses to each item ranging from 0 (not at all) to 4 (all the time). ^26^ The total score is the sum of all responses (ranging from 0 to 40). The AAI has demonstrated appropriate psychometric characteristics. ^26^ The AAI’s cut-off score for BDD is 19.

#### Hoarding Symptoms

The Hoarding Rating Scale-Self Report (HRS-SR) is a six-item instrument based on the original interview.^27,28^ The HRS-SR evaluates severity of clutter, difficulty discarding, excessive acquisition, distress, and impairment.^28^ Each item (structured as questions) can generate scores ranging from 0 (none) to 8 (extreme). Total scores include the summation of all responses, ranging from 0 to 40. The HRS-SF has demonstrated adequate psychometrics properties. ^28^ Sensitivity and specificity analyses indicate that the HRS-SR has a total clinical cutoff score of 14.^27^

#### Hair pulling

The Massachusetts General Hospital Hairpulling Scale (MGHHS) ^29^ is a seven-item self-report instrument that quantifies the severity of hair pulling in the previous week by assessing urges to pull hair, time spent pulling, perceived control, and distress associated with pulling. In the MGHHS, each item is scored on a 5-point Likert-scale from 0 (no symptoms) to 4 (severe symptoms). Items scores are summed to produce a total score (range 0 to 28), with a higher score indicating greater severity of hair pulling The MGHHS has shown acceptable psychometric features.^29^ A cut-off score of 17 for clinical significance has been suggested for the MSHHS.^30^

#### Skin Picking

The Skin Picking Scale-Revised (SPS-R) ^31^ is an eight-item self-report instrument that quantified the severity of skin picking in the previous week by assessing urges to pick skin (frequency/intensity), time spent, control, distress, interference, avoidance, and damage associated with skin picking. In the SPS-R, each item is scored on a 5-point Likert-scale ranging from 0 (no symptoms) to 4 (severe symptoms). Items scores are summed to produce a total score (range 0 to 24), with a higher score indicating greater severity of skin picking. The SPS-R has shown acceptable psychometric features. ^31^ A cut-off score of 9 for clinical significance has been suggested. ^30^

#### Psychological Distress

The Depression Anxiety Stress Scale - 21 (DASS-21) is a 21-item self-report questionnaire, based on the original 42-item scale, that quantifies negative affective experiences in the past week. ^32^ Respondents are asked to rate how much a specific statement applies to them using a 4-point Likert-scale that varies from 0 (‘did not apply to me at all’) to 3 (‘applied to me very much’). The DASS-21 generates three different subscores, namely, depression, anxiety, and stress. A total score is obtained by summing all subscales. The DASS-21 has shown excellent psychometric properties in a variety of contexts.^33^

#### Schizotypal Personality Symptoms

The Schizotypal Personality Questionnaire-Brief (SPQ-B) is a self-report questionnaire that lists 22 yes-or-no questions addressing different aspects of schizotypal personality. ^34^ Its total score is the sum of responses to items grouped in three different subscales, namely: interpersonal deficits (“People sometimes find me aloof and distant”), cognitive perceptual deficits (“Have you even had the sense that some person or force is around you, even though you cannot see anyone?”) and disorganization (“People sometimes comment on my unusual mannerisms and habits”). The SPQ-B total scores range from 0 to 22, with a higher score indicating greater severity of schizotypal symptoms. Initial regression models considered just the total scores. The SPQ-B has shown appropriate psychometric properties.^34^

#### Statistical analyses

A K-Means cluster analysis was performed using standardized values of the total BIS-11 and CHI-T scores. Two predefined number of clusters (three and four) were chosen based on the available literature describing “compulsive” and “impulsive” ends ^9^ or “compulsive”, “impulsive”, and “mixed” (four factors) ^10^ clusters in the general population. Further, regardless of the solution, we expected to identify am additional healthy group, which would be particularly relevant to be used reference for subsequent multinomial regression analysis (see below). A maximum of 20 iterations was chosen.

Subsequently, a multinomial regression models was planned using the categories above. The regression was performed with the cluster membership (“compulsive, “impulsive”, and “mixed”) as the dependent variables, and the DOCS, AAI, SPQ-B, HRS-SR, MGHHS and SPS-R as the independent (covariates) variables, also controlling for psychological distress and schizotypal symptoms. Then, an exploratory multinomial regression models were performed also using the cluster membership as the dependent variable but including the DOCS and SPQ-B subscores (instead of the DOCS and SPQ-B totals) plus the previous OCRDs scales as independent variables. The adopted level of significance was set at .05.

## Results

### Descriptive statistics

The initial sample comprised 829 participants, but 55 participants did not complete the CHI-T and/or the BIS-11. Thus, the final sample comprised 774 participants (53.1% females). Participants declared residing in the US in 99.9% of cases (in 0.1%, the information regarding origin was missing). Mean age was 38.70 (SD 12.73) years (minimum 18 and maximum 82 years). The majority of the sample was white (72.7%), had at least college education (91.1%), and was employed (89.3%). Participants declared a married status in 45.1% of the cases. Most participants (55.7% of the sample) reported not having a history of a previous mental illness diagnosis, and in 54.5% of the cases no family history of mental illness was reported.

### Cluster analyses

**Figure 1:**
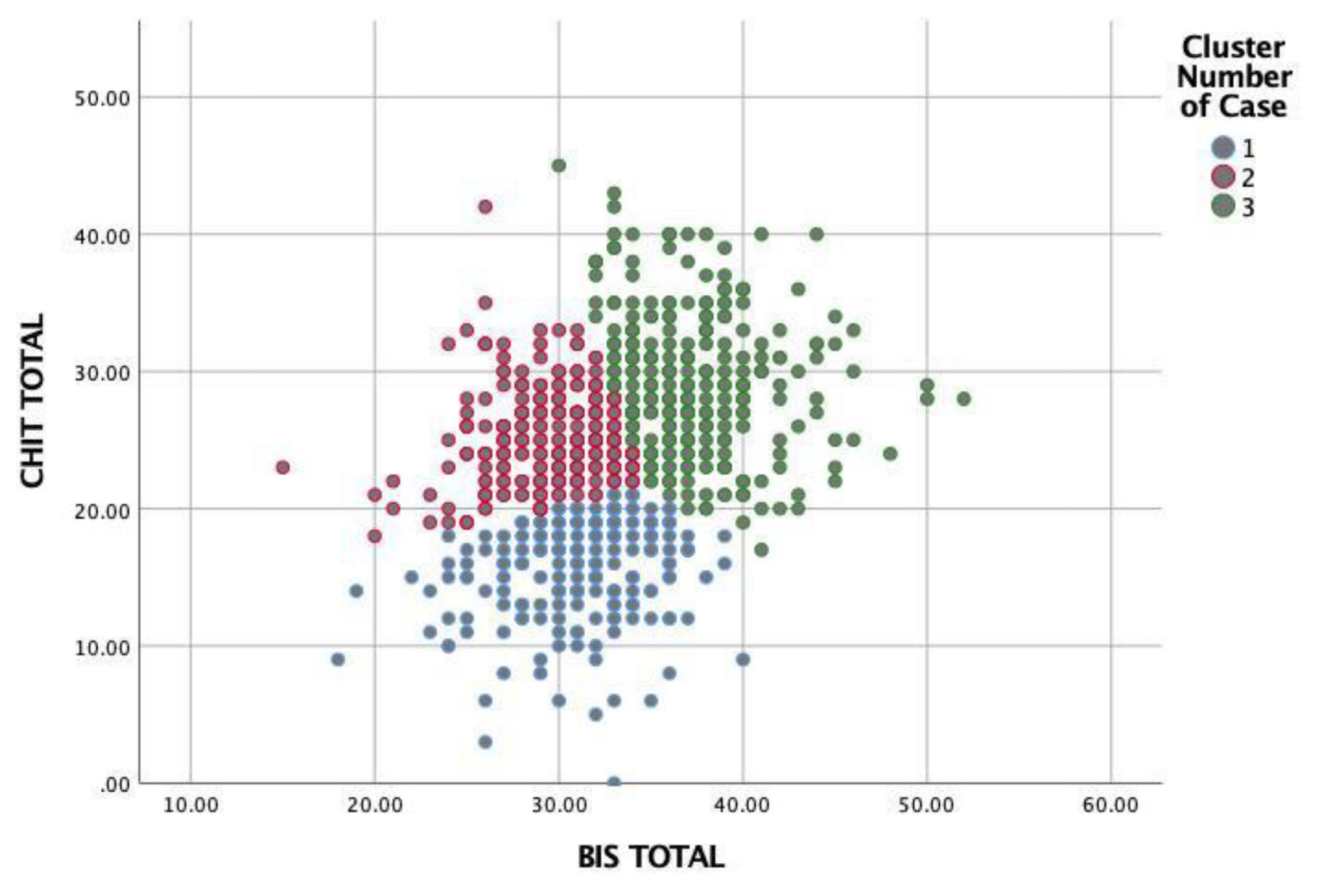
Scatterplot describing a three-cluster solution of the data with a mixed compulsive-impulsive (green) cluster, a predominantly compulsive (red) cluster and a healthy (blue) cluster.

The first solution, including three clusters (Figure 1A), generated one group low in both impulsivity and compulsivity (cluster 1, also termed the “healthy” group; n=203); one group high in compulsivity and low in impulsivity (cluster 2, also termed the “compulsive group”; n=278), and one group high in both compulsivity and impulsivity (cluster 3, also termed the “mixed group”; n=293) (Figure 1B). The number of iterations of this model to achieve convergence was eight and the minimum distance between initial centers was 5.07.

**Figure 2:**
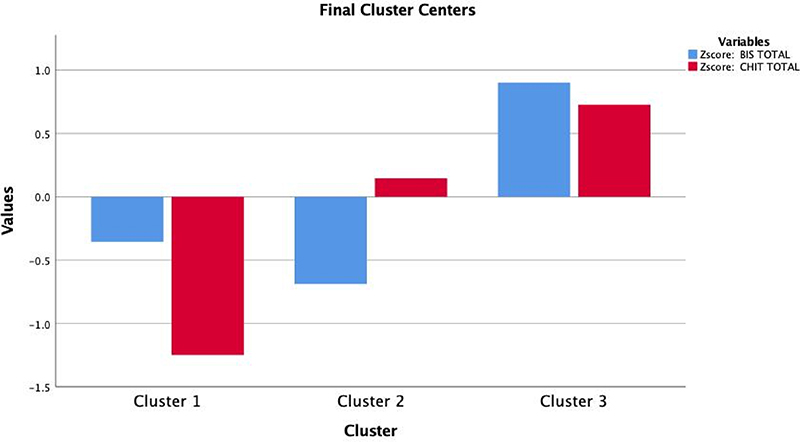
K-means clustering from the three-cluster solution revealing individuals low in compulsivity and low in impulsivity (cluster 1), high in compulsivity and low in impulsivity (cluster 2) and high in impulsivity and high in compulsivity (cluster 3).

A second solution, including four clusters, was also tested (Figure 2A). This model generated a high compulsivity and low impulsivity cluster (cluster 1, also termed “compulsive”; n=249); a high compulsivity and high impulsivity cluster (cluster 2, also termed “mixed”; n=181), a low compulsivity and low impulsivity cluster (cluster 3, also termed “healthy”; n=152) and a low compulsivity and high impulsivity cluster (cluster 4, also termed “impulsive”; n=192). Convergence was achieved after 18 iterations. The minimum distance between initial centers was 3.96.

Both first and second solutions were valid for showing significant and widespread differences between the groups in terms of severity of compulsivity and impulsivity (see appendix). Nevertheless, despite the greater number of iterations, the second solution was chosen for further testing for yielding more meaningful groups, for being more consistent with the theory of the compulsive-impulsive spectrum, and for including a group low on compulsivity and impulsivity thought to represent a healthier individuals. The last group was considered ideal for use as a reference category in the comparison with the other groups in a multinomial regression.

### Multinomial regression analyses

Regression models based on the four-cluster-solution were performed. The first regression model, using total scores of OCRDs constructs (plus DASS-21 and SPQ-B) as independent variables, was statistically significant (p < 0.001; LR χ2 = 150.35; log likelihood =1931.86). All Variance Inflation Factor levels were acceptable (below 5). As seen in table 1, it was found that (1) obsessive-compulsive symptoms (total DOCS scores), body dysmorphic symptoms (total AAI scores) and schizotypal symptoms (total SPQ-B scores) predicted the “compulsive” cluster, (2) that hoarding symptoms (HRS-SR scores) and depression, anxiety, and stress symptoms (DASS-21) predicted, on trend levels, the “impulsive” cluster and (3) that obsessive-compulsive symptoms (total DOCS scores), body dysmorphic symptoms (total AAI scores) and schizotypal symptoms (total SPQ-B scores) predicted the mixed cluster.

Nevertheless, because the predictors of the “compulsive” and the “mixed” clusters were essentially the same in the first regression model, a second regression model, which broke down DOCS and SPQ-B into their subscores, was also performed (Table 2). All Variance Inflation Factor levels were acceptable. This model was also statistically significant (p < 0.001; LR χ2 = 175.70; log likelihood =1907.90) and found that (1) more severe symmetry symptoms (DOCS subscores), body dysmorphic symptoms (total AAI scores) and SPQ-B interpersonal subscores predicted the “compulsive” cluster, (2) that the HRS-SR scores predicted (on a trend level), the “impulsive cluster” and that (3) symmetry symptoms (DOCS subscores), body dysmorphic symptoms (total AAI scores) and SPQ-B cognitive-perceptual subscores predicted the “mixed” cluster.

## Discussion

In this study which included 774 participants, we performed two K-means cluster analyses to investigate how individuals could be classified within a compulsive-impulsive spectrum. The solution most consistent with our hypothesis identified one “healthy” and three “clinical” (more severe) clusters (i.e. one mixed and more severe compulsive-impulsive group, one predominantly compulsive group and one predominantly impulsive group). This analysis was followed by two regressions to identify which OCRDs symptoms predicted these clusters (using the “healthy” group as a reference). Findings can be summarized in three main points. Firstly, as hypothesized, the compulsive cluster was predicted by OCD symptoms and (although unexpectedly) also by BDD and psychotic-like/schizotypal symptoms. Secondly, consistently with our hypothesis, BDD predicted the mixed cluster. However, similarly to the compulsive cluster, the later cluster was also associated with OCD and SCZ-like symptoms, instead of HD symptoms. Thirdly, the impulsive cluster was not predicted by TTM and SPD but, only marginally, by HD symptoms. Taken together, these findings indicate that OCRD symptoms (particularly OCD and BDD) do not map onto specific ends of the impulsive-compulsive spectrum, but that they are associated with high levels of mixed compulsive and impulsive traits.

OCD and BDD symptoms predicting the same clusters is consistent with comorbidity studies and other statistical models showing these disorders loading in the same factor. ^35^ On the other hand, the relationship between OCD and two clusters that are characterized by different impulsivity levels dovetails with the heterogeneous findings in the OCD literature ^36–44^ and suggest that the association between OCD and increased impulsivity might reflect the inclusion of specific OCD subpopulations (or phenotypes) in some studies. This interpretation is consistent with studies showing that impulsivity levels in OCD may be associated with distinct age at onset ^38,41^ and comorbidity patterns ^38,41,43–45^ In the present study, we were unable to confirm the findings by Stein et al., ^46^ who described aggression and sexual/religious symptoms in association with increased impulsivity in OCD patients. Arguably, the relationship between OCD symptoms and mixed compulsive and impulsive traits may also depend on the study setting, as individuals with compulsive traits who display increased impulsivity or unacceptable urges may be over-represented in treatment seeking samples for being more fearful of their symptoms.

We also found that BDD symptoms predicted membership to the same clusters as OCD did, namely, the mixed (and severe) cluster and the predominantly compulsive cluster. This finding is consistent with studies showing BDD individuals to exhibit increased compulsive and impulsive traits,^47^ higher prevalence of impulse control disorders (such as skin picking disorder^48^ and compulsive sexual behavior^47^), greater rates of alcohol and other substance use disorders,^49^ increased neurocognitive impulsivity,^50^ and increased suicide attempts and ideation ^51^. On the other hand, they also indicate that a significant amount of individuals with BDD may be related to compulsivity that is relatively devoid of associated impulsivity traits. Whether these later findings suggest that BDD symptoms with lower impulsivity levels may be better conceptualized as an obsessional form of BDD similar to the traditional Y-BOCS “somatic obsessions”, an “inhibited” (non-impulsive) form of BDD characterized by avoidant behaviour, or a “genuine” form of dysmorphia, remains to be clarified by future studies. It would be interesting to test how BDD symptoms within these clusters differ from each other.

Since OCD, BDD and psychotic-like symptoms predicted both compulsive and mixed clusters in a very similar way in our first regression model, we broke down OCD and SPQ-B total scores and tested, in a different exploratory model, whether their subscores were able to predict different compulsive and/or impulsive clusters. These analyses demonstrated that, in addition to OCD and BDD, the interpersonal schizotypal subscore was associated with the compulsive cluster, while the cognitive- perceptual schizotypy subscore predicted the mixed compulsive-impulsive group. Previous research has indicated that the interpersonal domain did not discriminate schizotypal personality disorder (SCZPD) from other personality disorders.^52^ Thus, our finding may simply indicate that interpersonal problems related to a number of conditions may be overrepresented in the predominantly compulsive cluster. In contrast, the cognitive-perceptual symptoms (namely, ideas of reference, odd beliefs, and perceptual disturbances), which show high sensitivity and moderate positive predictive value in terms of diagnosis of SCZPD,^52^ may reflect the more severe nature of the compulsive-impulsive cluster, which may also contain more psychotic- like symptoms.

In contrast to Snorrason et al^35^ who reported HD to load in an “obsessive-compulsive” factor (comprising OCD, BDD, and HD symptoms), we found HD symptoms to be associated, on a trend level, with a separate impulsive cluster. Despite not reaching statistical significance, this finding is consistent with the almost universal presence of acquisition behaviors (e.g. compulsive buying) in HD, which are conceptually related to impulsive behaviors, and the increased prevalence of hoarding symptoms in adults with attention deficit and hyperactivity disorder.^53^ Perhaps compulsive traits are not critical components of HD, and difficult discarding is more related to avoidance rather than compulsivity itself. Although it is difficult to explain this unanticipated finding (we initially hypothesized hoarding to predict the mixed cluster), we speculate it being to the lower numbers in the impulsive cluster and/or to the relatively short nature of the instrument to assess hoarding, which included only one broad item for inability to discard, thus not being able to fully capture the phenomenology complexity of HD. Finally, the fact that TTM and SPD symptoms did not emerge as independent predictors of the impulsive cluster might be ascribed to them being overshadowed by other highly associated constructs, particularly BDD. ^54^

Our study has a number of limitations including (1) its cross sectional nature, (2) the use of self-report instruments (i.e. BIS-11 and CHI-T) that either ignore important domains associated with impulsive behaviors (e.g. negative urgency) or that it is still new (despite promising results), (3) the lack of information on underlying cognitive functioning, and (4) the fact that compulsivity and impulsivity may shift to one another in a temporally dynamic manner.^10^ Also, though the sample was an online cohort expected to be normative, relatively high levels of symptoms were found. Future studies should investigate the stability of these clusters and whether individuals can transit between clusters, e.g. it is important to clarify if individuals from the impulsive or the compulsive traits cluster can convert to the compulsive-impulsive cluster under specific circumstance. Finally, our findings also suggest that increased compulsivity and impulsivity traits that are observed in the general population may increase the risk for OCRDs under exposure to stressful life events. Perhaps treatments targeting these traits in people exposed to other risk factors and that treatments targeting these traits should be pursued in the future with a view to decrease conversion to more severe OCRD symptoms.

## Figures and Tables

**Figure 3:**
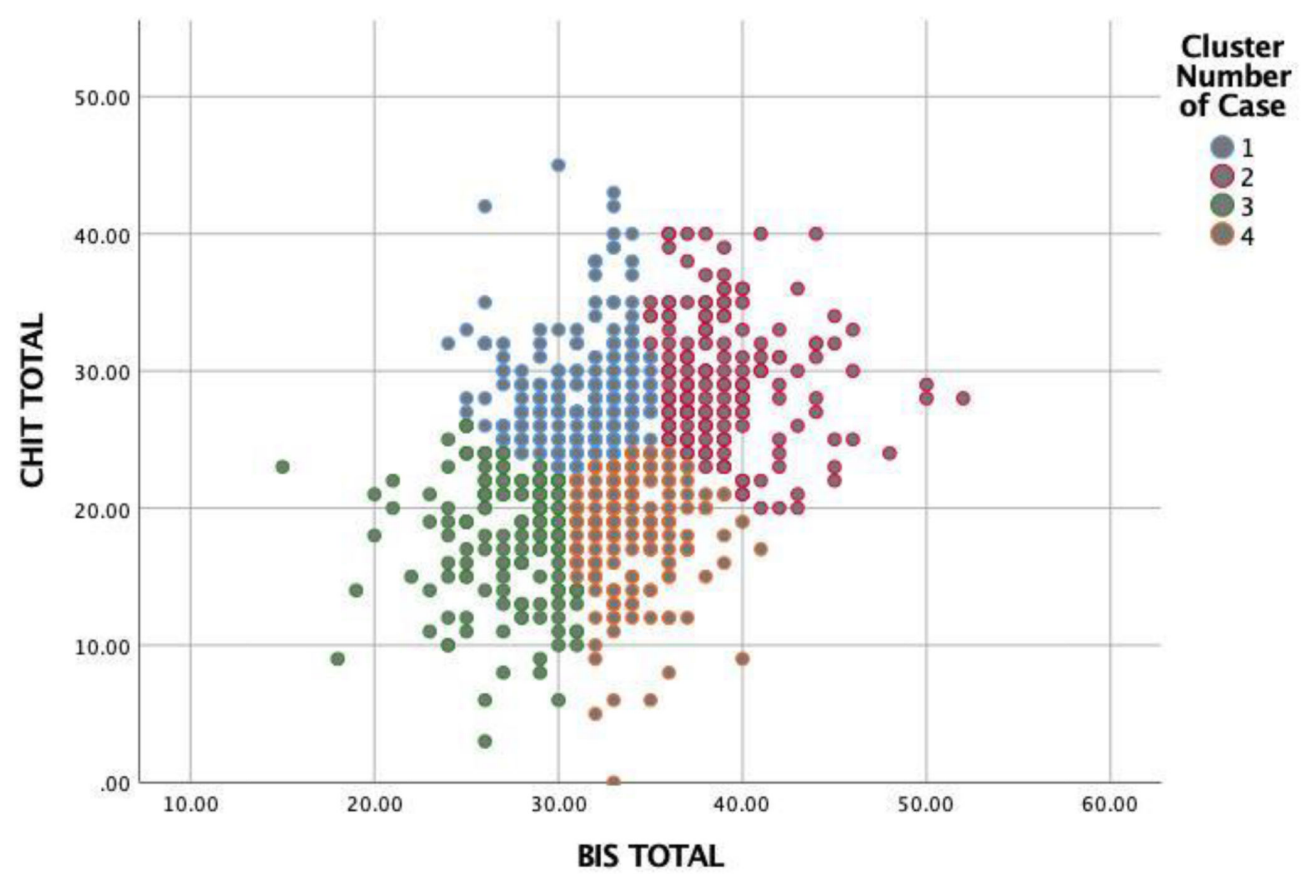
Scatterplot describing a four-cluster solution of the data with a mixed compulsive-impulsive (red) cluster, a predominantly compulsive (blue) cluster a predominantly impulsive (orange), cluster and a “healthy” (green) cluster.

**Figure 4:**
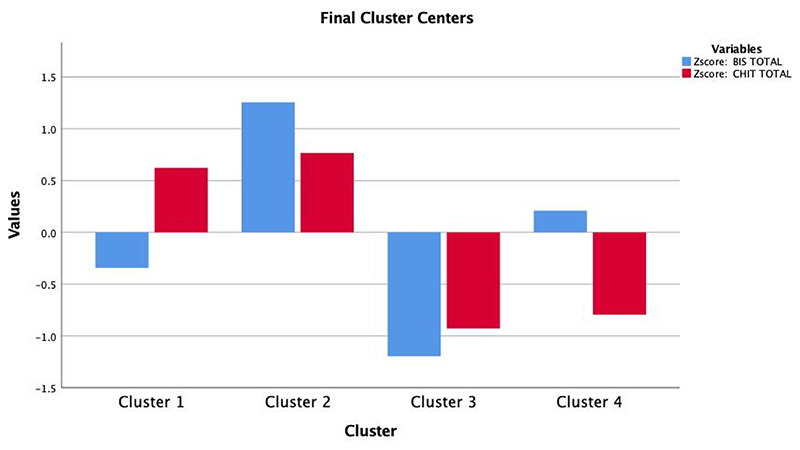
K-means clustering from the four-factor solution? revealing individuals high in compulsivity (cluster 1), high in compulsivity and impulsivity (cluster 2), low in compulsivity and in impulsivity (cluster 3, “healthy”), and high in impulsivity and low in compulsivity (cluster 4).

**Table 1:** First multinomial regression analysis describing the predictors of clusters 1 (compulsive), 2 (compulsive-impulsive) and 4 (impulsive).

Cluster number								95% Confidence interval for Exp (B)	
		B	Std.Error	Wald	df	Sig.	Exp(B)	Lower bound	Upper bound
1									
	(Intercept)	-.566	.203	7.759	1	.005			
	**DOCS**	**.031**	**.011**	**8.413**	**1**	.004*	**1.031**	**1.010**	**1.053**
	**AAI**	**.047**	**.021**	**5.153**	**1**	.023*	**1.048**	**1.006**	**1.091**
	HRS	-.005	.019	.068	1	.795	.995	.958	1.033
	MGH-HPS	-.004	.036	.011	1	.915	.996	.928	1.069
	SPD R	.002	.030	.003	1	.958	1.002	.944	1.063
	**SPQ-B**	**.066**	**.025**	**6.797**	**1**	.009*	**1.068**	**1.017**	**1.123**
	DASS 21	-.017	.014	1.483	1	.223	.983	.957	1.010
									
2									
	(Intercept)	-1.65	.244	45.653	1	.000			
	**DOCS**	**.029**	**.011**	**6.326**	**1**	.012*	**1.029**	**1.006**	**1.052**
	**AAI**	**.076**	**.021**	**12.991**	**1**	**<. 001**	**1.079**	**1.035**	**1.125**
	HRS	.007	.020	.107	1	.743	1.007	.968	1.047
	MGH-HPS	.034	.036	.918	1	.338	1.035	.965	1.110
	SPD R	.032	.031	1.055	1	.304	1.032	.972	1.096
	**SPQ-B**	**.071**	**.027**	**6.602**	**1**	.010*	**1.073**	**1.017**	**1.133**
	DASS 21	-.005	.015	.116	1	.734	.995	.967	1.024
									
4									
	(Intercept)	.046	.199	.054	1	.816			
	DOCS	.015	.011	1.753	1	.186	1.015	.993	1.038
	AAI	.029	.023	1.677	1	.195	1.030	.985	1.076
	HRS	-.036	.022	2.628	1	.105	.965	.924	1.007
	MGH-HPS	.020	.038	.284	1	.594	1.021	.947	1.100
	SPD R	.038	.032	1.422	1	.233	1.038	.976	1.105
	SPQ-B	.023	.027	.736	1	.391	1.023	.971	1.079
	DASS 21	-.028	.015	3.274	1	.070	.972	.943	1.002
									

Footnote: DOCS=Dimensional Obsessive-Compulsive Scale; AA= Appearance Anxiety Inventory; HRS=Hoarding Rating Scale; MGHHPS=Massachusetts General Hospital Hair Pulling Scale; SPD-R=Skin Picking Disorder-Revised, SPQ-B-Schizotypal Personality Questionnaire-Brief; and DASS- 21=Depression Anxiety and Stress Scale- 21 items.

**Table 2:** Second multinomial regression analysis describing the predictors of clusters 1 (compulsive), 2 (compulsive-impulsive) and 4 (impulsive).but including subscores of DOCS

Cluster number								95% Confidence interval for Exp (B)	
		B	Std.Error	Wald	df	Sig.	Exp(B)	Lower bound	Upper bound
1									
	(Intercept)	-.705	.248	8.106	1	.004			
	DOCS -fear of harm	.033	.040	.661	1	.416	1.033	.955	1.118
	DOCS - unacceptable thoughts	-.038	.040	.931	1	.335	.962	.890	1.040
	DOCS - contamination	.026	.031	.685	1	.408	1.026	.965	1.092
	**DOCS - symmetry**	**.123**	**.049**	**6.301**	**1**	.012*	**1.131**	**1.027**	**1.245**
	**AAI total**	**.045**	**.021**	**4.652**	**1**	.031*	**1.046**	**1.004**	**1.090**
	HRS	-.006	.019	.088	1	.767	.994	.957	1.033
	MGH-HPS	.000	.036	.000	1	.993	1.000	.932	1.074
	SPS-R	-.001	.031	.001	1	.981	.999	.941	1.062
	SPQ-B– cognitive perceptual	.092	.072	1.602	1	.206	1.096	.951	1.263
	**SPQ-B — interpersonal**	**.132**	**.054**	**6.024**	**1**	.014*	**1.141**	**1.027**	**1.268**
	SPQ -B– disorganized	-.029	.084	.116	1	.733	.972	.825	1.145
	DASS 21	-.015	.014	1.086	1	.297	.985	.958	1.013
									
2									
	(Intercept)	-1.32	.281	22.215	1	.000			
	DOCS –fear of harm	.049	.045	1.194	1	.275	1.050	.962	1.146
	DOCS - unacceptable thoughts	-.025	.043	.343	1	.558	.975	.897	1.060
	DOCS - contamination	-.018	.035	.249	1	.617	.983	.917	1.053
	**DOCS - symmetry**	**.120**	**.052**	**5.356**	**1**	.021*	**1.127**	**1.019**	**1.248**
	**AAI total**	**.075**	**.022**	**12.207**	**1**	<.001*	**1.078**	**1.034**	**1.125**
	HRS	.004	.020	.046	1	.830	1.004	.965	1.045
	MGH-HPS	.028	.036	.607	1	.436	1.029	.958	1.104
	SPS-R	.021	.031	.441	1	.507	1.021	.960	1.085
	**SPQ-B— cognitive perceptual**	**.162**	**.077**	**4.498**	**1**	.034*	**1.176**	**1.012**	**1.367**
	SPQ-B– interpersonal	-.015	.061	.061	1	.806	.985	.875	1.110
	SPQ-B – disorganized	.106	.091	1.367	1	.242	1.112	.931	1.329
	DASS 21	-.002	.015	.017	1	.897	.998	.969	1.028
									
4									
	(Intercept)	-.028	.239	.014	1	.905			
	DOCS –fear of harm	-.037	.043	.753	1	.386	.963	.885	1.048
	DOCS - unacceptable thoughts	.013	.043	.085	1	.771	1.013	.931	1.102
	DOCS - contamination	.043	.032	1.801	1	.180	1.044	.980	1.113
	DOCS - symmetry	.053	.054	.970	1	.325	1.054	.949	1.171
	AAI total	.026	.023	1.339	1	.247	1.027	.982	1.073
	*HRS*	*-.036*	*.022*	*2.697*	*1*	*.101*	*.964*	*.924*	*1.007*
	MGH-HPS	.018	.038	.219	1	.640	1.018	.945	1.097
	SPS-R	.036	.032	1.270	1	.260	1.037	.974	1.104
	SPQ-B– cognitive perceptual	.067	.077	.750	1	.386	1.069	.919	1.242
	SPQ-B– interpersonal	.007	.055	.018	1	.894	1.007	.904	1.122
	SPQ-B– disorganized	.022	.089	.061	1	.805	1.022	.859	1.216
	DASS 21	-.025	.016	2.482	1	.115	.975	.946	1.006

Footnote: DOCS=Dimensional Obsessive-Compulsive Scale; AA= Appearance Anxiety Inventory; HRS=Hoarding Rating Scale; MGHHPS=Massachusetts General Hospital Hair Pulling Scale; SPD-R=Skin Picking Disorder-Revised, SPQ-B- Schizotypal Personality Questionnaire-Brief; and DASS-21=Depression Anxiety and Stress Scale- 21 items.
